# The Influence of Time of Day of Vaccination with BNT162b2 on the Adverse Drug Reactions and Efficacy of Humoral Response against SARS-CoV-2 in an Observational Study of Young Adults

**DOI:** 10.3390/vaccines10030443

**Published:** 2022-03-14

**Authors:** Paweł Matryba, Karol Gawalski, Iga Ciesielska, Andrea Horvath, Zbigniew Bartoszewicz, Jacek Sienko, Urszula Ambroziak, Karolina Malesa-Tarasiuk, Anna Staniszewska, Jakub Golab, Rafał Krenke

**Affiliations:** 1 Department of Immunology, Medical University of Warsaw, 02-097 Warsaw, Poland; karol.gawalski@student.wum.edu.pl (K.G.); jgolab@wum.edu.pl (J.G.); 2 The Doctoral School of the Medical University of Warsaw, Medical University of Warsaw, 02-097 Warsaw, Poland; 3 Department of Internal Medicine, Pulmonary Diseases and Allergy, Medical University of Warsaw, 02-097 Warsaw, Poland; iga.markowska@student.wum.edu.pl (I.C.); karolina.malesa-tarasiuk@wum.edu.pl (K.M.-T.); rkrenke@wum.edu.pl (R.K.); 4 Department of Pediatrics, Medical University of Warsaw, 02-097 Warsaw, Poland; andrea.horvath@wum.edu.pl; 5 Department of Internal Medicine and Endocrinology, Medical University of Warsaw, 02-097 Warsaw, Poland; zbigniew.bartoszewicz@wum.edu.pl (Z.B.); uambroziak@wum.edu.pl (U.A.); 6 2nd Department of Obstetrics and Gynecology, Medical University of Warsaw, 02-097 Warsaw, Poland; jacek.sienko@wum.edu.pl; 7 Department of Experimental and Clinical Pharmacology, Medical University of Warsaw, 02-097 Warsaw, Poland; astaniszewska@wum.edu.pl

**Keywords:** COVID-19, SARS-CoV-2, circadian rhythm, vaccination, BNT162b2, adverse drug reactions, humoral response, vaccine efficacy, time of day, young adults

## Abstract

An increasing body of evidence from both academic and clinical studies shows that time-of-day exposure to antigens might significantly alter and modulate the development of adaptive immune responses. Considering the immense impact of the COVID-19 pandemic on global health and the diminished efficacy of vaccination in selected populations, such as older and immunocompromised patients, it is critical to search for the most optimal conditions for mounting immune responses against SARS-CoV-2. Hence, we conducted an observational study on 435 healthy young adults vaccinated with two doses of BNT162b2 (Pfizer-BioNTech) vaccine to determine whether time-of-day of vaccination influences either the magnitude of humoral response or number of adverse drug reactions (ADR) being reported. We found no significant differences between morning and afternoon vaccination in terms of both titers of anti-Spike antibodies and frequency of ADR in the studied population. In addition, our analysis of data on the occurrence of ADR in 1324 subjects demonstrated that the second administration of vaccine in those with previous SARS-CoV-2 infection was associated with lower incidence of ADR. In aggregate, vaccination against COVID-19 with two doses of BNT162b2 mRNA vaccine is presumed to generate an equally efficient anti-Spike humoral response.

## 1. Introduction

Decades of continued vaccination programs against infectious diseases such as polio, tuberculosis, diphtheria, tetanus, pertussis, measles, mumps and rubella have established their undeniable efficiency, as well as their major role in the sustainability of healthcare systems worldwide [1]. The recent COVID-19 pandemic [2] clearly highlights the importance of development and wide availability of vaccination programs for sustainability of current expectancy and quality of human life. Hence, substantial efforts are being put into generation of strategies for the improvement of vaccine efficacy and efficiency. So far, one of the leading fields is the development of novel, more sophisticated adjuvants [3], usually targeting dendritic cell functions [4]. However, in parallel to the rapid progress done in chemistry research of adjuvant design, behavioral aspects such as quality of sleep [5] and its deprivation [6], exercising [7,8,9,10,11], smoking [12] and a proper nutritional status with regard to dietary fiber [13], vitamins [14] and other micronutrients [15] have started to emerge as a potent approach capable of modifying the magnitude of immune response upon vaccination [12].

Another promising path towards the improvement of vaccine efficacy is related to findings in the field of circadian rhythms of the immune system. Although, for many years, it has been widely recognized that significant fluctuations in the function of the innate immune system exists [16], such as time-of-day dependent amount of cytokine release by tissue macrophages upon LPS stimulation [17,18] or neutrophil recruitment [19], recent experiments shed new light on the potential impact of circadian rhythms on adaptive immune responses [20]. Notably, as many as 6% of all protein-coding transcripts in murine CD8^+^ T cells are in sync with the magnitude of their response to vaccination under modulation of the molecular clock [21]. Interestingly, Druzd et al. [22] showed that severity of experimental autoimmune encephalitis was associated with the time of immunization (zeitgeber, ZT, 8 vs. ZT20) with worse outcomes found in ZT8-immunized mice. This effect, however, was abrogated in T cell-specific *Bmal1*^−/−^ mice. 

Data on whether the time-of-day difference in the exposure to antigens during vaccination results in a varying efficacy of mounting of the adaptive immune responses are very scarce and in humans consists of as little as ~10 trials [23,24,25,26,27,28,29,30,31,32]. The limited number of existing trials, along with their heterogeneity in terms of both age, overall health status of participants and studied vaccines (against influenza, hepatitis A and B, tuberculosis [20]), lead to the mixed results in regard to effectiveness of mounting humoral responses and characteristics of adverse drug reactions (ADR) between morning versus afternoon vaccinations. So far, two studies have assessed immunological response against SARS-CoV-2 in the context of circadian rhythms. Zhang et al. [31] reported that morning administration of the inactivated SARS-CoV-2 vaccine (BBIBP-CorV, Sinopharm’s Beijing Institute of Biological Products, Beijing, China) to a group of 63 young (aged 24–28) healthcare workers resulted in a two-fold increase of titers of neutralizing antibodies. On the contrary, Wang et al. [32] studied 2784 participants and observed superiority of vaccination (in terms of anti-Spike antibodies titers) with both Pfizer mRNA and AstraZeneca adenoviral vaccines during afternoon vaccination 2 weeks after the first dose of vaccine.

Considering the severity of the COVID-19 pandemic impact on global health, limited efficacy of vaccines in groups of e.g., older [33], dialyzed [34], immunocompromised [35] or patients with hematologic malignancies [36] and evident need for additional vaccine doses in otherwise healthy subjects [37], it is critical to search for the most optimal conditions for mounting the immune response against SARS-CoV-2 [38]. To this aim, we collected data on ADR from >1000 young adult individuals and assessed the relationship between the time-of-day of vaccination and IgG levels against COVID-19 anti-Spike protein (anti-S) in patients reporting reliable data on the time-of-day of vaccination. 

## 2. Materials and Methods

### 2.1. Study Design

**Figure 1:**
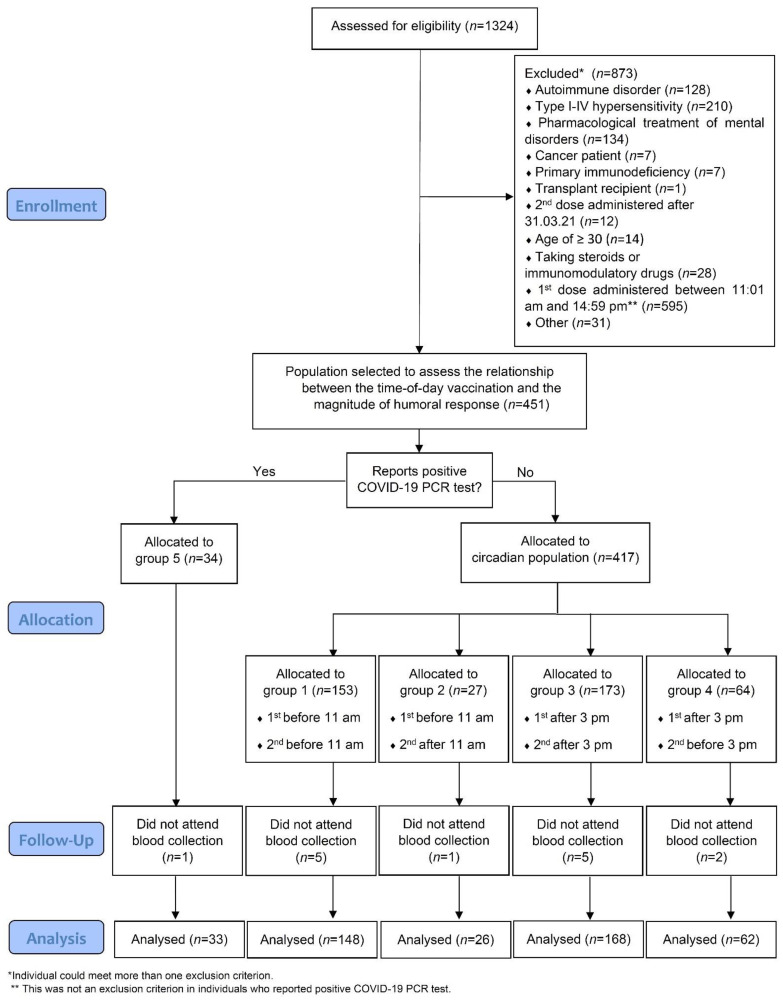
CONSORT diagram of participant enrollment and retention throughout the study.

This was a cross-sectional study involving vaccinated students, with two doses of the BNT162b2 COVID-19 vaccine (Pfizer-BioNTech, Pfizer Inc., New York, NY, USA), attending the Medical University of Warsaw, Poland. First, an online anonymous questionnaire was used to collect data regarding health and vaccination status, ADR, positive COVID-19 PCR, date and time-of-day of vaccinations and self-assessed chronotype. The online survey was available from 27 May 2021 to 7 June 2021. All students who completed the questionnaire were included in the analysis of ADR. Then, a specific sub-population was selected to determine whether time-of-day of vaccination was associated with the magnitude of humoral response. The process of selection and allocation to subgroups based on the time-of-day of vaccination is depicted in Figure 1. The major exclusion criterion in the process of patient selection for humoral response assessment was the time of vaccination with the first dose between 11:01 am and 2:59 pm (*n* = 595). 

### 2.2. Eligibility and Exclusion Criteria for the Circadian Population 

In order to be enrolled in the study of anti-Spike antibody levels (circadian section of the study) through the initial questionnaire, a 2-dose vaccination regimen had to be completed before 1 April 2021, with the first dose administered before 11 am or after 3 pm. Students that reported e.g., autoimmune disease, diagnosis of cancer or immunodeficiency, current treatment with steroids or other immunosuppressive or immunomodulatory drugs, current pregnancy and transplant recipients were excluded from the circadian section of the study. This resulted in the selection of 451 eligible students; 435 (96.45%) had blood collected for the measurement of anti-Spike antibody level. The second vaccine dose was administered to eligible participants between 21 January and 31 March, with 92.87% participants receiving their second dose before 13 of February. Before data analysis, students from the circadian part of the study were divided into 4 (1–4) groups based on the time of administration of the vaccine. 

### 2.3. Blood Collection

Blood collection was performed in the afternoon (1–4 pm) between 7 and 17 of June 2021 at Independent Public Central Clinical Hospital, Warsaw, Poland. Blood was collected in tubes (#02.1063.001, Sarstedt, Numbrecht, Germany) and clotted at room temperature before centrifugation at 1500× *g* for 15 min. The separated serum was frozen and kept at −80 °C for later analysis. 

### 2.4. Measurement of Antibodies

IgG and IgM antibodies directed against the nucleocapsid (N) protein were measured in the sera of patients using high sensitive electrochemiluminescence qualitative sandwich immunoassay Elecsys Anti-SARS-CoV-2 (#09203095190, Roche Diagnostics, Basel, Switzerland) and analyzed with an automatic immunodiagnostic analyzer Cobas 411e (Roche Diagnostics).

The concentration of IgG antibodies with neutralizing properties directed against the S1 domain of the S protein were determined by the highly sensitive manual anti-SARS-CoV-2 QuantiVac ELISA sandwich immunoassay (#E1 2606-9601-10G, Euroimmun, Lübeck, Germany). This test is standardized against the WHO standard (NIBSC code: 20/136) and allows for the test results to be presented in international units: BAU/mL (BAU = binding antibody units). The color intensities of individual wells were measured using an automatic 8-channel ELISA LEDETECT 96 plate reader (Biomed, Salzburg, Austria) with photometric reading, equipped with LED lamps, at a wavelength of 450 nm with a 620 nm cut-off filter using MikroWin 2010/2013 software (Mikrotek Laborsysteme GmbH, Overath, Germany). Ascent Software (Ver. 2.6) from Labsystems, (Helsinki, Finland) was used for analysis of the results.

### 2.5. Statistical Analysis

Data analysis was carried out using R software, versions 4.0.5/4.1.2. Nominal variables were presented with count (*n*) and with % frequency. Continuous variables were presented as means ± SD with range or median (Q1; Q3) with range depending on normality of distribution. Distribution normality was verified using the Shapiro–Wilk test, as well as the skewness and kurtosis values and visual assessment of histograms. Comparison of anti-S COVID-19 antibodies between groups was made using ANOVA analysis or independent *t*-test, as appropriate. Analysis of the number of ADR between groups was made with Mann–Whitney U test or Kruskal–Wallis test. For comparisons of two groups, MD (mean or median difference) between groups with 95% confidence interval (CI) was calculated. In case of significant Kruskal–Wallis test outcome, Dunn’s test was used as post hoc evaluation. Due to some violation of parametric tests assumptions (e.g., unequal subgroups’ size), all parametric tests were repeated with non-parametric equivalents, which confirmed results of parametric analysis. Analysis of correlation between continuous variables was made using Spearman’s correlation coefficient. Time between anti-S antibodies measurement and the second dose was calculated as the number of days. Comparisons of % frequencies between groups were performed with a chi-square Pearson test or Fisher exact test. All analyses were based on significance level α = 0.05.

## 3. Results

### 3.1. Incidence of ADR Related to the BNT162b2 COVID-19 Vaccine in Young Adults

Initially, we examined the characteristics of ADR from all 1324 respondents to provide the preliminary detailed data on their prevalence in a group of young adults (an overview of participants is presented in Table 1).

As expected, the number of ADR was significantly higher after the second dose (Table 2) after which 75% of individuals reported four or fewer ADR when compared with the first vaccination, that caused two or fewer ADR in 75% of subjects (we relate only to the number, not to severity of ADR, as no grade 3 or higher ADR were observed).

Since women experienced more ADR after the first vaccine dose than men, we decided to expand the characterization of ADR after first and second dose grouped by sex. As presented in Table 3, the prevalence of ADR between men and women did not differ, besides frequency of headache after the first and the second dose and arthralgia after the second dose—with men being at 35%, 20% and 40% lower risk for developing these symptoms, respectively.

### 3.2. Number of ADR Does Not Depend on the Time-of-Day of Vaccination

In order to assess the main aim of this study, i.e., a possible impact of time-of-day of vaccination on the magnitude of humoral response and ADR, we further examined 435 young and healthy individuals of Caucasian race (an overview of participant characteristics is presented in Table 4) from the previously described group of 1324 questionnaire responders, hereinafter named as the “circadian population”. The circadian population included individuals who have been vaccinated with (i) two doses of BNT162b2 COVID-19 vaccine (Pfizer-BioNTech) before 1 April 2021, (ii) first dose before 11 am or after 3 pm and (iii) met additional criteria such as lack of immune system disorder or treatment with steroids (please see Materials and Methods section for precise description).

First, we aimed to assess if morning vs afternoon vaccination might result in a difference in the number of ADR. Due to the scarce literature existing on this topic, we assumed that all scenarios are probable i.e., time of the first, second or both vaccinations might impact the number of ADR. It turned out that, although clear tendency existed towards more frequent reporting of ADR in groups vaccinated in the morning (Table 5), during the (1) first, (2) second and (3) both administrations of vaccine, the level of statistical significance was not reached (*p* = 0.107; 0.051 and 0.054, respectively).

Interestingly, participants tested as anti-N positive reported statistically fewer ADR after the second dose than those who were anti-N negative at the time of blood collection (Table 6). This might imply that we observe fewer ADR after boosters of BNT162b2 COVID-19 vaccine. 

### 3.3. Time-of-Day of Vaccination Does Not Alter Levels of Anti-S Antibody

Assuming that the time of the first administration of vaccine might determine the magnitude of developing humoral response, we compared the anti-S levels between those vaccinated with the first dose before 11 am (groups 1 and 2) and after 3 pm (groups 3 and 4). Although the mean levels of anti-S antibody were higher under every studied combination in the morning groups (all participants, all participants excluding anti-N positive and participants divided based on sex), the observed differences did not reach statistical significance (Table 7). Next, we performed a more detailed analysis based on time of both the first and second dose of vaccination (groups 1 to 4) or reported COVID-19 positive PCR result (group 5), again seeing no statistically significant differences between designated groups (Table S1). Moreover, no differences between anti-S levels and chronotypes (owl, early bird, no preference) reported by the participants in the questionnaire were observed (Table S2).

### 3.4. Past Infection with COVID-19 Elicits a Stronger Humoral Immune Response upon Vaccination

Out of 435 participants who were selected for blood collection as a circadian section, 33 (7.6%) reported to have at least one COVID-19 positive PCR test result, of whom 31 were tested positive before the first dose, 1 in-between doses and 1 after the second dose. In order to assess the number of symptom-free cases and use them for further analyses, we performed qualitative measurements of IgG and IgM antibodies directed against the COVID-19 N protein. Interestingly, we detected that in the PCR-negative group (402 subjects), 65 participants (16.2%) were N-positive. It should be underlined, however, that not every PCR-positive individual was detected as N-positive. In groups of 31 PCR-positive before first dose, 1 in-between doses and 1 after second dose, 25, 1 and 0 participants were N-positive, respectively. As expected, N-positive individuals (both women and men) elicited stronger anti-S response following 2-dose vaccination (Table 8). Importantly, this effect was not influenced by the time between administration of the second vaccine dose and blood collection in all studied groups (mean time 124.4 days with standard deviation of 8.7 days, Table S3).

## 4. Discussion

An increasing body of evidence from both academic and clinical studies shows that time-of-day exposure to antigen might significantly alter and modulate the development of adaptive immune responses [20]. Indeed, circadian rhythmicity of dendritic-, T- and B-cells was reported on every level of their function, starting from development [20,39] through trafficking [22,40,41], ending on activation [21,42,43] and exhaustion [44]. Recently, Holtkamp et al. [41] indicated that migration of DCs into skin lymphatics is rhythmic and under the direct control of the circadian clock gene *BMAL1*. Taking the pivotal role of DCs during mounting of immune responses into account and intradermal/intramuscular administration of the majority of existing vaccines, this underlines the possible importance of studying circadian rhythms in search for the best immunization strategy. The undeniable role of present and upcoming vaccines and vaccine-like formulations in both prevention and treatment of infectious diseases, cancer and even chronic diseases, makes the optimal activation of the immune system a vital issue for healthcare systems around the globe. 

In this study, we first aimed to provide a detailed list of ADR occurrences in a group of young adults (*n* = 1324 individuals). Clearly, the most frequently reported ADR after the first dose were pain at the injection site, headache and muscle pain, a list that was accompanied by fever and chills after the second dose. Interestingly, no differences between the frequency of ADR were reported between females and males, except for headache and arthralgia, which occurred more often in women. Lack of grade 3 or higher ADR abrogated the analysis of severity of ADR in addition to their occurrence. 

Since circadian rhythms observed in the immune system might not only affect the immune response as presented by the titers of antibodies [45], but also the strength and occurrence of immediate and late ADR [46], we asked whether number and characteristics of the reported ADR correlate with time-of-day of vaccination. So far, Langlois et al. [26] and Pollmann and Pollmann [24] have presented that local ADR tend to be more frequent in individuals revaccinated late during the daytime. In the case of BNT162b2, although we saw a clear tendency, the occurrence of ADR was not significantly higher in individuals vaccinated in the morning vs afternoon. It should be noted, however, that the most frequently observed ADR, such as pain and reddening at injection site, headache or fever, occur within a few hours after vaccination and tend to rapidly resolve. Hence, the difference, even if significant, could be at least to some extent caused by the mentioned delay in onset of symptoms, not time of vaccination. What is intriguing is the fact that individuals tested positive against COVID-19 nucleocapsid presented fewer ADR after the second dose than those who were detected as anti-N negative. Assuming that the observed difference is related to the number of times a particular individual was exposed to COVID-19 Spike protein, this might result in better tolerance of booster doses.

Finally, we showed that time of the vaccine administration does not impact the levels of COVID-19 anti-S antibodies. A difference was not observed independently of whether time of the first or both doses were used to group circadian population. Until now, two studies on the topic of circadian influence on COVID-19 vaccinated individuals have been performed by Zhang et al. [31] and Wang et al. [32], presenting mixed results, i.e., superiority of morning and afternoon vaccination, respectively. The discrepancy between our trial and Zhang et al. [31] might be due to examination of different vaccine types, i.e., mRNA-based BNT162b2 (Pfizer-BioNTech) and inactivated BBIBP-CorV (Sinopharm) and, possibly, limited number of participants (*n* = 63) in the latter study. On the other hand, Wang et al. [32] reported that afternoon vaccination with Pfizer-BioNTech elicits stronger humoral responses, however the measurements were done only after administration of the first dose and the effect seems to diminish significantly over time when groups with blood collected 2 weeks and 6 weeks after vaccination are compared. Another reason that might explain the lack of time-of-vaccination effect in our study relates to the molecular dynamics of Spike protein expression. Of note, in other circadian studies that used inactivated vaccines against influenza [25], hepatitis A [27] and tuberculosis [30] (and subunit vaccine against hepatitis B [28]), the full load of antigen is present at a time of vaccine administration. This is not the case with BNT162b2 vaccine, in which mRNA translation (expression of antigen) is spread over time that might diminish the presumed effect of daytime. Recently, Kurupati et al. [47] provided evidence that concentration of neutralizing antibodies might not be correlated to a time of the day when vaccination occurred, but rather time of blood collection—our study does not include such bias as blood was collected within a strict time frame of 3 h (1 pm to 4 pm) within 10 days. 

There are several limitations of our study that could be addressed in later trials. First, we collected blood only after a full (2-dose) vaccination regimen, thus potentially omitting the daytime effect after the first dose observed by Wang et al. [32]. Moreover, blood was not collected after a fixed number of days after complete vaccination (e.g., 100 days), but after a flexible amount of time with a little variation (with mean time between vaccination and blood collection of 124.4 days with standard deviation of 8.7 days). Lastly, it is disputable whether a study on a group of older individuals, with less efficient immune system [48,49], could lead to more informative results. On the one hand, a more efficient immune system of young adults could mask the influence of circadian rhythms, on the other hand circadian rhythms tend to wane with aging [50].

## 5. Conclusions

It should be concluded that time of vaccination does not impact the magnitude of humoral response in young adults ~4 months after full vaccination against COVID-19 with BNT162b2 mRNA vaccine, nor cause significant differences in either frequency or severity of reported ADR. As the level of neutralizing antibodies is not the only determinant of protection against SARS-CoV-2, we suggest that information regarding time of vaccination should be further tested in large-scale studies to address the correlation between circadian rhythms, chronotype and risk reduction of infection in humans.

## Figures and Tables

**Table 1:** Characteristics of the group.

	*n*	% of Group	Mean ± SD /Median (Q1;Q3)	Range
N	1324	100.0		
Sex, female	959	77.7		
Age at first dose, years	1320		23.34 ± 0.05	20–37
Body mass index (BMI)	1324		21.86 ± 0.09	15.79–45.51
COVID-PCR positive before first dose	57	4.6		
COVID-PCR positive between first and second dose	3	0.2		
COVID-PCR positive after second dose	3	0.2		
Cancer	7	0.5		
Autoimmune disease	128	9.7		
Type I-IV hypersensitivity disease	210	15.9		
Primary immunodeficiency	7	0.5		
Transplant recipient	1	0.1		
Taking steroids or other immunomodulatory drugs	28	2.1		
Pharmacological treatment of mental disorders	134	10.1		
Alcohol overuse	10	0.8		

**Table 2:** Comparison of a number of ADR between first and second dose.

	Studied Timepoint/Sex		MD (95% CI)	*p*
	**First Dose**	**Second Dose**		
Number of ADR (all participants)	1.00 (0.00;2.00)	1.00 (0.00;4.00)	0.00 (0.0000;0.0000)	**<0.001**
	**Females**	**Males**		
Number of ADR after first dose	1.00 (0.00;2.00)	1.00 (0.00;1.00)	0.00 (0.0000;0.0000)	**0.040**
Number of ADR after second dose	1.00 (0.00;4.00)	1.00 (0.00;3.00)	0.00 (−0.0001;0.0000)	0.059

Data are presented as medians (Q1;Q3), groups are compared with Mann–Whitney U test. MD—median difference between groups with 95% confidence interval.

**Table 3:** Comparison of frequency of ADR between males and females for selected symptoms.

	Females	Males	RR (95% CI)	*p*
**ADR after first dose** **, *n* (%)**				
Pain at the injection site	571 (59.5%)	202 (55.3%)	0.93 (0.84;1.03)	0.186
Reddening at the injection site	67 (7.0%)	27 (7.4%)	1.06 (0.69;1.63)	0.888
Elevated body temperature (>37.5 °C)	59 (6.2%)	23 (6.3%)	1.02 (0.64;1.63)	>0.999
Chills	69 (7.2%)	21 (5.8%)	0.80 (0.50;1.28)	0.418
Headache	125 (13.0%)	31 (8.5%)	0.65 (0.45;0.95)	**0.028**
Muscle pain	140 (14.6%)	40 (11.0%)	0.75 (0.54;1.04)	0.102
Arthralgia	54 (5.6%)	16 (4.4%)	0.78 (0.45;1.34)	0.442
Cough	5 (0.5%)	0 (0.0%)	n/a	0.378
Dyspnea	2 (0.2%)	0 (0.0%)	n/a	0.935
Insomnia	35 (3.6%)	10 (2.7%)	0.75 (0.38;1.50)	0.518
Tiredness	22 (2.3%)	9 (2.5%)	1.07 (0.50;2.31)	>0.999
Nausea	9 (0.9%)	2 (0.5%)	0.58 (0.13;2.69)	0.718
Lymphadenopathy	22 (2.3%)	14 (3.8%)	1.67 (0.86;3.23)	0.176
Diarrhea	7 (0.7%)	2 (0.5%)	0.75 (0.16;3.60)	>0.999
**ADR after second dose** **, *n* (%)**				
Pain at the injection site	494 (51.5%)	177 (48.5%)	0.94 (0.83;1.06)	0.357
Reddening at the injection site	60 (6.3%)	30 (8.2%)	1.31 (0.86;2.00)	0.252
Elevated body temperature (>37.5 °C)	248 (25.9%)	78 (21.4%)	0.83 (0.66;1.03)	0.105
Chills	262 (27.3%)	82 (22.5%)	0.82 (0.66;1.02)	0.084
Headache	291 (30.3%)	89 (24.4%)	0.80 (0.65;0.99)	**0.038**
Muscle pain	307 (32.0%)	98 (26.8%)	0.84 (0.69;1.02)	0.079
Arthralgia	144 (15.0%)	33 (9.0%)	0.60 (0.42;0.86)	**0.006**
Cough	7 (0.7%)	4 (1.1%)	1.50 (0.44;5.10)	0.751
Dyspnea	7 (0.7%)	2 (0.5%)	0.75 (0.16;3.60)	>0.999
Insomnia	72 (7.5%)	30 (8.2%)	1.09 (0.73;1.65)	0.750
Tiredness	41 (4.3%)	19 (5.2%)	1.22 (0.72;2.07)	0.562
Nausea	20 (2.1%)	3 (0.8%)	0.39 (0.12;1.32)	0.181
Lymphadenopathy	71 (7.4%)	17 (4.7%)	0.63 (0.38;1.05)	0.095
Diarrhea	10 (1.0%)	2 (0.5%)	0.53 (0.12;2.39)	0.600

Groups compared with chi-square Pearson test. RR—relative risk (reference = female) with 95% confidence interval.

**Table 4:** Characteristics of the circadian population.

	*n*	% of Group	Means ± SD /Median (Q1;Q3)	Range
Number of participants	435	100.0		
Sex, female	331	76.1		
Age at first dose, years	435		23.25 ± 1.79	20–29
BMI	435		21.71 ± 3.26	16.11–45.42
Anti-N, positive (>1)	91	20.9		
Anti-S, BAU/mL × 1000	435		102.92 ± 59.97	3.58–323.00
**First dose time**				
Before 11 am	182	41.8		
11:01 am–2:59 pm	16	3.7		
After 3 pm	237	54.5		
**Second dose time**				
Before 11 am	173	39.8		
11:01 am–2:59 pm	83	19.1		
After 3 pm	179	41.1		
COVID-PCR positive before first dose	31	7.1		
COVID-PCR positive between first and second dose	1	0.2		
COVID-PCR positive after second dose	1	0.2		
Part-time job during the night	12	2.8		
Part-time job during the night for at least 2 weeks with at least 3 night shifts per week	4	0.9		
**Declared chronotype**				
No preferences	107	24.6		
Early bird (usually sleeps between 11 pm–7 am)	169	38.9		
Night owl (usually sleeps between 2–10 am)	159	36.6		
Experienced at least 1 ADR after first dose	263	60.5		
Pain at the injection site	255	58.6		
Reddening at the injection site	33	7.6		
Elevated body temperature (>37.5 °C)	27	6.2		
Chills	23	5.3		
Headache	51	11.7		
Muscle pain	59	13.6		
Arthralgia	13	3.0		
Cough	2	0.5		
Dyspnea	0	0.0		
Diarrhea	4	0.9		
Insomnia	16	3.7		
Lymphadenopathy	11	2.5		
Anaphylaxis	0	0.0		
Acute peripheral facial palsy	0	0.0		
Experienced at least 1 ADR after second dose	262	60.2		
Pain at the injection site	217	49.9		
Reddening at the injection site	26	6.0		
Elevated body temperature (>37.5 °C)	100	23.0		
Chills	107	24.6		
Headache	121	27.8		
Muscle pain	136	31.3		
Arthralgia	60	13.8		
Cough	6	1.4		
Dyspnea	4	0.9		
Diarrhea	3	0.7		
Insomnia	33	7.6		
Lymphadenopathy	39	9.0		
Anaphylaxis	0	0.0		
Acute peripheral facial palsy	0	0.0		
Number of ADR after first dose	494	n/a	1.00 (0.00;2.00)	0–12
Number of ADR after second dose	852	n/a	1.00 (0.00;4.00)	0–11

**Table 5:** Comparison of number of ADR between patients who received first dose before 11:00 and after 15:00.

	Studied Population		MD (95% CI)	*p*
	**Group 1 + 2**	**Group 3 + 4**		
Number of ADR after first dose	1.00 (0.00;2.00)	1.00 (0.00;2.00)	0.00 (0.0000;0.0000)	0.319
Number of ADR after second dose	2.00 (0.00;4.00)	1.00 (0.00;4.00)	−1.00 (0.0000;0.0000)	0.107
	**second dose before11 am**	**second dose after 3 pm**		
Number of ADR after second dose	2.00 (0.00;4.00)	1.00 (0.00;4.00)	−1.00 (−0.0001;0.0000)	0.051
	**Group 1**	**Group 3**		
Number of ADR after second dose	2.00 (0.00;4.00)	1.00 (0.00;4.00)	−1.00 (−0.0001;0.0000)	0.054

Data are presented as medians (Q1;Q3), groups are compared with Mann-Whitney U test. MD—median difference between groups with 95% confidence interval.

**Table 6:** Comparison of number of ADR between anti-N positive and anti-N in norm patients.

	Anti-N Positive	Anti-N in Norm	MD (95% CI)	*p*
Number of ADR after first dose	1.00 (0.00;2.00)	1.00 (0.00;2.00)	0.00 (0.0000; 0.0001)	0.092
Number of ADR after second dose	0.00 (0.00;3.00)	1.50 (0.00;4.00)	1.50 (0.0000; 1.0000)	**0.001**

Data presented as median (Q1;Q3), groups are compared with Mann–Whitney U test. MD—median difference between groups with 95% confidence interval.

**Table 7:** Comparison of anti-S antibody levels between groups based on time of the first vaccination.

	Group 1 + 2	Group 3 + 4	MD (95% CI)	*p*
All	104.73 ± 60.41	99.53 ± 59.19	5.20 (−6.63;17.03)	0.388
All excl. anti-N positive	98.92 ± 57.48	92.11 ± 52.57	6.81 (−5.12;18.75)	0.262
Females	103.57 ± 60.64	97.11 ± 54.01	6.46 (−6.66;19.58)	0.333
Females excl. anti-N positive	98.97 ± 58.10	90.61 ± 48.96	8.36 (−4.92;21.63)	0.216
Males	108.76 ± 60.20	106.40 ± 71.94	2.36(−24.23;28.95)	0.861
Males excl. anti-N positive	98.77 ± 56.00	96.85 ± 63.00	1.92 (−25.39;29.23)	0.889

Data are presented as means ± SD, groups are compared with independent *t*-test. MD—mean difference between groups with 95% confidence interval. Antibody units are presented as BAU/mL × 1000.

**Table 8:** Comparison of anti-S antibody levels between anti-N groups.

	Anti-N Positive	Anti-N in Norm	MD (95% CI)	*p*
All	133.32 ± 69.76	94.87 ± 54.44	38.45 (22.85;54.05)	**<0.001**
Females	132.43 ± 67.69	94.08 ± 52.85	38.35 (20.33;56.37)	**<0.001**
Males	135.43 ± 75.75	97.62 ± 59.91	37.81 (5.22;70.40)	**0.024**

Data are presented as means ± SD, groups are compared with independent *t*-test. MD—mean difference between groups with 95% confidence interval. Antibody units are presented as BAU/mL × 1000.

## Data Availability

The datasets generated during and/or analyzed during the current study are available upon request.

## Supplementary Materials

The following supporting information can be downloaded at: https://www.mdpi.com/article/10.3390/vaccines10030443/s1, Table S1: Comparison of anti-S antibody levels between studied groups; Table S2: Comparison of anti-S level between reported lifestyle groups; Table S3: Correlation of anti-S level with the time between anti-S level measurement and administration of the second dose.
