# Supplementary material for: The Influence of Time of Day of Vaccination with BNT162b2 on the Adverse Drug Reactions and Efficacy of Humoral Response against SARS-CoV-2 in an Observational Study of Young Adults
Source: Vaccines (Basel). 2022 Mar 14;10(3):443. doi: 10.3390/vaccines10030443 (PMC8954058; doi:10.3390/vaccines10030443)
Supplement: Supplementary file 1 [file vaccines-10-00443-s001.zip › vaccines-1617850-supplementary.pdf]

vaccines

MDPI

Supplementary tables

Table S1. Comparison of anti-S antibody levels between studied groups.

	Group 1	Group 2	Group 3	Group 4	Group 5	p
All	102.48±58.32	117.54±71.06	99.98±59.41	98.33±59.41	117.86±62.58	0.808
All excl. anti-N positive	95.24±53.93	118.93±72.02	90.34±49.24	96.50±60.30	80.54±15.17	0.555
Females	100.39±57.26	122.96±77.23	98.26±54.53	94.35±53.18	119.45±66.26	0.759
Females excl. anti-N positive	93.83±52.81	127.20±77.17	90.29±47.08	91.36±53.73	80.54±15.17	0.438
Males	110.06±62.38	102.81±52.88	104.28±70.62	114.88±79.73	109.60±42.78	0.976
Males excl. anti-N positive	100.61±58.76	89.18±42.52	90.49±55.98	114.88±79.73	-	0.873

Data are presented as means±SD, groups are compared with ANOVA. Antibody units are presented as BAU/ml x 1000.

Table S2. Comparison of anti-S level between reported lifestyle groups.

	Early birds	Owls	No preferences	p
All	96.98±54.80	108.96±64.44	103.32±60.55	0.194
All excl. anti-N positive	89.42±49.44	99.90±54.22	95.58±62.03	0.293
Females	98.14±53.90	104.92±61.75	102.41±59.42	0.650
Females excl. anti-N positive	91.94±48.42	97.19±53.60	93.14±59.10	0.771
Males	92.18±58.99	118.29±70.05	106.68±65.79	0.219
Males excl. anti-N positive	78.08±53.40	106.59±55.89	106.16±74.79	0.157

Data presented as means±SD, groups are compared with ANOVA.

Table S3. Correlation of anti-S level with the time between anti-S level measurement and administration of the 2nd dose.

	All		Females		Males	
	rho	p	rho	p	rho	p
All	-0.04	0.397	-0.03	0.645	-0.08	0.392
Group 1	0.08	0.311	0.02	0.824	0.24	0.177
Group 2	-0.38	0.056	-0.34	0.150	-0.56	0.192
Group 3	-0.06	0.474	-0.02	0.792	-0.10	0.483
Group 4	-0.07	0.565	0.00	0.994	-0.12	0.712
Group 5	-0.14	0.455	0.00	0.993	-0.87	0.054
Anti-N positive	0.16	0.120	**0.25**	**0.049**	-0.04	0.856
Anti-N negative	-0.08	0.124	-0.08	0.192	-0.10	0.368

rho - Spearman correlation coefficient.
